# Prognostic Impact of Primary Tumor Extent and Postoperative Radiation Facility Location in Major Salivary Gland Malignancies

**DOI:** 10.7759/cureus.24038

**Published:** 2022-04-11

**Authors:** Colton Ladbury, Jason Liu, Rebecca Nelson, Arya Amini, Ellie Maghami, Sagus Sampath

**Affiliations:** 1 Radiation Oncology, City of Hope National Medical Center, Duarte, USA; 2 Biostatistics, City of Hope National Medical Center, Duarte, USA; 3 Head and Neck Surgery, City of Hope National Medical Center, Duarte, USA

**Keywords:** radiation oncology, postoperative radiation therapy, ncdb, salivary gland malignancy, big data

## Abstract

Introduction

The treatment of primary salivary malignancies often requires a multimodality approach. The purpose of this analysis was to evaluate the interaction between primary tumor extent and the treatment location of postoperative radiotherapy (PORT) in patients with primary salivary malignancies with respect to survival outcomes.

Methods

Patients with primary salivary malignancies who underwent upfront surgery followed by radiation were queried in the National Cancer Database (NCDB). Patients were stratified by pathologic T stage and whether PORT was performed at the same or different facility as the definitive surgery. Survival outcomes were compared using the Kaplan-Meier method and Cox proportional hazards regression.

Results

A total of 5,553 patients were selected, of which 1,159 had pathologic T4 (pT4) tumors. Patients who received PORT at the same facility compared with a different facility demonstrated superior overall survival (OS) on log-rank analysis (p=0.003). On subgroup analysis, patients with pT4 tumors had superior OS (p=0.015), whereas patients with smaller T1-3 tumors did not. PORT receipt at the same surgical facility was not a significant predictor of OS on multivariable analysis when all patients were included (p=0.057). However, among patients with pT4 tumors, OS was improved in patients who got PORT at the same facility as their surgery (p=0.015), with 10-year survival rates of 38.3 (95% confidence interval (CI): 33%-44%) versus 31% (95%CI: 24%-38%).

Conclusion

OS was improved in patients with primary salivary malignancies who received PORT at the same facility as their surgery, but the difference appears to be primarily driven by patients with pT4 primary tumors.

## Introduction

The definitive treatment of malignancies arising from major salivary glands primarily consists of complete surgical resection [1], when possible, which may be potentially curative as a single modality. However, adverse clinical and pathologic features may warrant postoperative radiotherapy (PORT), including intermediate- to high-grade histologic variants, advanced T stage, close or positive surgical margins, tumor spillage, lymphovascular space invasion, neural/perineural invasion, and nodal involvement [1]. For patients who require multimodality treatment, direct communication among the treatment team is critical in determining plans for adjuvant therapy, as well as optimal timing [2]. Furthermore, high-risk regions need to be carefully mapped out in association with the surgeon, especially in more advanced tumor stages with distorted anatomy.

Because the definitive courses of PORT for salivary tumors often last more than six weeks and require daily treatment, due to convenience considerations (such as a shorter drive for daily treatments) and referral patterns, among other factors, patients may receive their radiation at a facility other than their initial surgical facility. However, due to the complexity of head and neck cancer treatment, there is evidence that more experienced high-volume centers specializing in the treatment of head and neck cancers using both surgery and radiation produce superior outcomes [3-5]. Considering the value of cancer center expertise and the interplay between surgical resection and radiation treatment planning, we hypothesized that patients who require more extensive surgical resection, as often occurs in patients with higher T stage, would have improved outcomes if their PORT was performed at the same facility as their surgical facility. Therefore, we performed a retrospective analysis using the National Cancer Database (NCDB) to assess the prognostic impact of receiving surgery and PORT at the same facility, with stratification by T stage, to help inform recommendations for adjuvant treatment.

## Materials and methods

Patient demographics and treatment variables

The NCDB is a joint project of the Commission on Cancer of the American College of Surgeons and the American Cancer Society. It is a hospital-based registry that represents 70% of all cancer cases in the United States, drawing data from more than 1,500 commission-accredited cancer programs [6]. The NCDB contains detailed information on disease stage, risk factors specific to head and neck cancer, and receipt of treatment including surgery, radiation, and chemotherapy delivered during the first course of treatment. The data used in the study are derived from a deidentified NCDB file. The American College of Surgeons and the Commission on Cancer have not verified and are not responsible for the analytic or statistical methodology employed or the conclusions drawn from these data by the investigator. The NCDB has established criteria to ensure the data submitted meets specific quality benchmarks. The current analysis was performed with the approval of the City of Hope institutional review board under protocol number 22164.

We initially queried patients with major salivary gland carcinomas (all histologies) diagnosed between 2004 and 2017. Patients with unknown pathologic T or N staging or metastatic disease were excluded. The included histologies were subsequently limited to adenoid cystic carcinoma (histology code: 8200), mucoepidermoid carcinoma (histology code: 8430), acinic cell carcinoma (histology code: 8550), carcinoma-ex-pleomorphic carcinoma (histology code: 8941), adenocarcinoma (histology codes: 8140-8190, 8255-8311, 8480-8482, and 8570-8575), and salivary duct carcinoma (histology code: 8500) [7]. Patients included in the primary query received upfront definitive surgery at the reporting facility and had known follow-up. Patients receiving neoadjuvant radiation therapy (RT) were excluded. The final group selected had known locations of RT, either at the same site of the reporting facility where upfront surgery was performed or at a site different from where the surgery was performed. Due to the hypothesis that the primary benefit would occur in patients with more advanced diseases, further analysis was performed on patients with a pathologic T4 (pT4) primary tumor.

Relevant patient and treatment characteristics were included, as coded in the NCDB. Each patient stage was based on the edition corresponding to their year of diagnosis (American Joint Committee on Cancer (AJCC) sixth or seventh edition). There were negligible differences between the staging of salivary malignancies from the AJCC sixth edition to the seventh edition. Treating facility type is defined based on the Commission on Cancer Accreditation program. Time from diagnosis to surgery and time from diagnosis to the start of RT were subtracted from one another to define the time between surgery and RT; the study defined PORT delay using a cutoff of 42 days (six weeks) based on the National Comprehensive Cancer Network (NCCN) guideline recommendations, suggesting that PORT starts within 5-6 weeks after surgery [1]. The NCDB records the elapsed days of RT from the start of RT to the finish, and the cutoff of 46 days (median) was selected for stratification. The primary endpoint was overall survival (OS). Disease-specific survival is not included in the NCDB and so could not be examined.

Statistical analysis

Statistical analyses were performed using open-source libraries in Python 3.8 (PSF, Wilmington, DE). The primary outcome was OS in patients stratified by facility at which the PORT was performed. OS interval was calculated from the date of diagnosis per the reporting institution to the date of death or last follow-up. Those alive at last contact were censored at their last follow-up date. Patient demographic, clinicopathologic, and treatment-related characteristics were used to describe the selected patient cohort. Analysis of these factors among the location of PORT treatment was conducted using χ2 tests for categorical variables and t-tests for continuous variables that were normally distributed. Mann-Whitney U statistics were calculated for continuous data that were not normally distributed. Univariate (UVA) and multivariable (MVA) logistic regression were performed to investigate factors associated with the receipt of PORT at a different facility. OS was first examined using the Kaplan-Meier method, with comparisons between cohorts using log-rank tests. Univariate survival analysis was performed with the log-rank test and unadjusted Cox proportional hazards models to estimate hazard ratios (HR). MVA Cox proportional hazards regression was also used for survival analysis to investigate the impact of the location of PORT after adjustment for potential covariates. All variables included in the descriptive statistics were included in the initial MVAs but were removed using backward stepwise selection.

## Results

**Figure 1:**
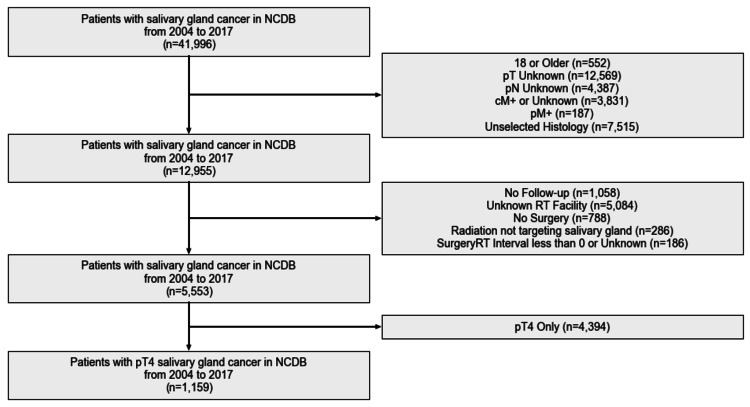
Patient selection schema illustrating exclusion criteria NCDB: National Cancer Database; pT: pathologic T stage; pN: pathologic N stage; cM: clinical M stage; pM: pathologic M stage; RT: radiation therapy

A total of 5,553 patients with salivary gland malignancies were identified, of which 1,159 had pathologic T4 disease. The full patient selection diagram is visualized in Figure 1. Among all patients, 74.4% of the rate of PORT being performed at the same facility as surgery was 74.4% among all patients and 71.4% among T4 patients. The median age of the cohort was 61 years (range: 18-90 years). Full descriptive statistics are listed in Table 1. A greater proportion of patients receiving PORT at a different facility were older than 65 years (47.7% versus 41.3%, p=0.052) and lived ≥25 miles from their surgical facility (47.4% versus 24.8%, p<0.001), and the number of Charlson/Deyo comorbidities were more often ≥2 (6.3% versus 3.8%, p=0.060). There were no differences in the proportion of patients with positive surgical margins (48.6% versus 52.7%, p=0.446) or with radiation initiated within six weeks (50.5% versus 53.3%, p=0.733).

**Table 1:** Patient and treatment characteristics by location of PORT treatment for patients with primary salivary malignancies LVSI: lymphovascular space invasion; ECE: extracapsular extension; RT: radiation therapy

	All Patients			T4 Patients		
Characteristic	Different Facility (%)	Same Facility (%)	p	Different Facility (%)	Same Facility (%)	p
Count	1,420 (25.6%)	4,133 (74.4%)		333 (28.7%)	826 (71.3%)	
Age (Years)			0.031			0.052
≤65	875 (61.6%)	2,680 (64.8%)		174 (52.3%)	485 (58.7%)	
>65	545 (38.4%)	1,453 (35.2%)		159 (47.7%)	341 (41.3%)	
Facility Volume			0.425			0.559
	733 (51.6%)	2,186 (52.9%)		143 (42.9%)	372 (45%)	
≥Median	687 (48.4%)	1,947 (47.1%)		190 (57.1%)	454 (55%)	
Sex			0.605			0.988
Female	684 (48.2%)	1,956 (47.3%)		145 (43.5%)	361 (43.7%)	
Male	736 (51.8%)	2,177 (52.7%)		188 (56.5%)	465 (56.3%)	
Race			0.008			0.472
Asian/Pacific Islander	38 (2.7%)	151 (3.7%)		7 (2.1%)	26 (3.1%)	
Black	143 (10.1%)	472 (11.4%)		27 (8.1%)	92 (11.1%)	
Hispanic	54 (3.8%)	235 (5.7%)		12 (3.6%)	40 (4.8%)	
Native American	5 (0.4%)	15 (0.4%)		2 (0.6%)	3 (0.4%)	
Other	8 (0.6%)	24 (0.6%)		1 (0.3%)	5 (0.6%)	
Unknown	10 (0.7%)	49 (1.2%)		3 (0.9%)	9 (1.1%)	
White	1,162 (81.8%)	3,187 (77.1%)		281 (84.4%)	651 (78.8%)	
Charlson/Deyo Score			0.004			0.06
0	1,129 (79.5%)	3,425 (82.9%)		257 (77.2%)	682 (82.6%)	
1	218 (15.4%)	564 (13.6%)		55 (16.5%)	113 (13.7%)	
2+	73 (5.1%)	144 (3.5%)		21 (6.3%)	31 (3.8%)	
Insurance			0.005			0.136
Government	693 (48.8%)	1,841 (44.5%)		183 (55%)	435 (52.7%)	
Private	677 (47.7%)	2,104 (50.9%)		138 (41.4%)	371 (44.9%)	
Uninsured	40 (2.8%)	121 (2.9%)		8 (2.4%)	7 (0.8%)	
Unknown	10 (0.7%)	67 (1.6%)		4 (1.2%)	13 (1.6%)	
Mean Zipcode Income ($)			0.003			0.07
63,000+	396 (27.9%)	1,352 (32.7%)		84 (25.2%)	265 (32.1%)	
≤62,999	876 (61.7%)	2,405 (58.2%)		215 (64.6%)	486 (58.8%)	
Unknown	148 (10.4%)	376 (9.1%)		34 (10.2%)	75 (9.1%)	
Region			<0.001			0.03
Midwest	357 (25.1%)	1,073 (26%)		97 (29.1%)	236 (28.6%)	
Northeast	508 (35.8%)	1,611 (39%)		116 (34.8%)	348 (42.1%)	
South	196 (13.8%)	389 (9.4%)		45 (13.5%)	72 (8.7%)	
Unknown	145 (10.2%)	511 (12.4%)		21 (6.3%)	62 (7.5%)	
West	214 (15.1%)	549 (13.3%)		54 (16.2%)	108 (13.1%)	
Hospital Type			0.007			0.09
Academic/Research Program	640 (45.1%)	1,754 (42.4%)		197 (59.2%)	433 (52.4%)	
Community Cancer Program	93 (6.5%)	219 (5.3%)		19 (5.7%)	32 (3.9%)	
Comprehensive Community Cancer Program	375 (26.4%)	1,218 (29.5%)		69 (20.7%)	215 (26%)	
Integrated Network Cancer Program	167 (11.8%)	431 (10.4%)		27 (8.1%)	84 (10.2%)	
Unknown	145 (10.2%)	511 (12.4%)		21 (6.3%)	62 (7.5%)	
Distance to Facility (Miles)			<0.001			<0.001
≤25	735 (51.8%)	2,885 (69.8%)		141 (42.3%)	547 (66.2%)	
>25	540 (38%)	878 (21.2%)		158 (47.4%)	205 (24.8%)	
Unknown	145 (10.2%)	370 (9%)		34 (10.2%)	74 (9%)	
Primary Site			0.466			0.866
Not Specified	36 (2.5%)	101 (2.4%)		8 (2.4%)	18 (2.2%)	
Parotid	1,237 (87.1%)	3,658 (88.5%)		304 (91.3%)	762 (92.3%)	
Sublingual	15 (1.1%)	32 (0.8%)		4 (1.2%)	6 (0.7%)	
Submandibular	132 (9.3%)	342 (8.3%)		17 (5.1%)	40 (4.8%)	
Margin Status			0.162			0.446
Negative	795 (56%)	2,193 (53.1%)		154 (46.2%)	349 (42.3%)	
Positive	565 (39.8%)	1,754 (42.4%)		162 (48.6%)	435 (52.7%)	
Unknown	60 (4.2%)	186 (4.5%)		17 (5.1%)	42 (5.1%)	
Grade			0.034			0.986
Grade 1	173 (12.2%)	573 (13.9%)		19 (5.7%)	47 (5.7%)	
Grade 2	297 (20.9%)	837 (20.3%)		56 (16.8%)	133 (16.1%)	
Grade 3 or 4	591 (41.6%)	1,570 (38%)		179 (53.8%)	443 (53.6%)	
Unknown	359 (25.3%)	1,153 (27.9%)		79 (23.7%)	203 (24.6%)	
LVSI			0.002			0.387
LVSI+	284 (20%)	714 (17.3%)		98 (29.4%)	230 (27.8%)	
LVSI-	596 (42%)	1,639 (39.7%)		108 (32.4%)	245 (29.7%)	
Unknown	540 (38%)	1,780 (43.1%)		127 (38.1%)	351 (42.5%)	
ECE			0.04			0.863
ECE+	153 (10.8%)	391 (9.5%)		57 (17.1%)	131 (15.9%)	
ECE-	765 (53.9%)	2,133 (51.6%)		155 (46.5%)	387 (46.9%)	
Unknown	502 (35.4%)	1,609 (38.9%)		121 (36.3%)	308 (37.3%)	
Lymph Node Dissection			0.002			0.069
Lymph Node Dissection	1,323 (93.2%)	3,727 (90.2%)		322 (96.7%)	775 (93.8%)	
No Lymph Node Dissection	92 (6.5%)	395 (9.6%)		11 (3.3%)	51 (6.2%)	
Unknown	5 (0.4%)	11 (0.3%)		0 (0%)	0 (0%)	
Histology			0.838			0.614
Acinic Cell Carcinoma	213 (15%)	670 (16.2%)		28 (8.4%)	80 (9.7%)	
Adenocarcinoma	316 (22.3%)	875 (21.2%)		96 (28.8%)	231 (28%)	
Adenoid Cystic Carcinoma	270 (19%)	772 (18.7%)		77 (23.1%)	197 (23.8%)	
Carcinoma-Ex-Pleomorphic Carcinoma	101 (7.1%)	275 (6.7%)		26 (7.8%)	44 (5.3%)	
Mucoepidermoid Carcinoma	432 (30.4%)	1,277 (30.9%)		80 (24%)	197 (23.8%)	
Salivary Duct Carcinoma	88 (6.2%)	264 (6.4%)		26 (7.8%)	77 (9.3%)	
Pathologic T Stage			<0.001			1
T1	354 (24.9%)	1,053 (25.5%)		--	--	
T2	347 (24.4%)	1,254 (30.3%)		--	--	
T3	386 (27.2%)	1,000 (24.2%)		--	--	
T4	333 (23.5%)	826 (20%)		333 (100%)	826 (100%)	
Pathologic N Stage			0.114			0.884
N0	891 (62.7%)	2,739 (66.3%)		155 (46.5%)	390 (47.2%)	
N1	177 (12.5%)	477 (11.5%)		49 (14.7%)	111 (13.4%)	
N2	346 (24.4%)	903 (21.8%)		126 (37.8%)	320 (38.7%)	
N3	6 (0.4%)	14 (0.3%)		3 (0.9%)	5 (0.6%)	
Time Between Surgery and RT (Weeks)			0.61			0.733
≤6	605 (42.6%)	1,795 (43.4%)		141 (42.3%)	339 (41%)	
>6	815 (57.4%)	2,338 (56.6%)		192 (57.7%)	487 (59%)	
Duration of RT (Days)			<0.001			<0.001
≤46	745 (52.5%)	2,333 (56.4%)		168 (50.5%)	440 (53.3%)	
>46	588 (41.4%)	1,739 (42.1%)		144 (43.2%)	371 (44.9%)	
Unknown	87 (6.1%)	61 (1.5%)		21 (6.3%)	15 (1.8%)	
Received Chemotherapy			0.465			0.096
No	1,143 (80.5%)	3,349 (81%)		243 (73%)	553 (66.9%)	
Unknown	34 (2.4%)	77 (1.9%)		6 (1.8%)	12 (1.5%)	
Yes	243 (17.1%)	707 (17.1%)		84 (25.2%)	261 (31.6%)	

**Figure 2:**
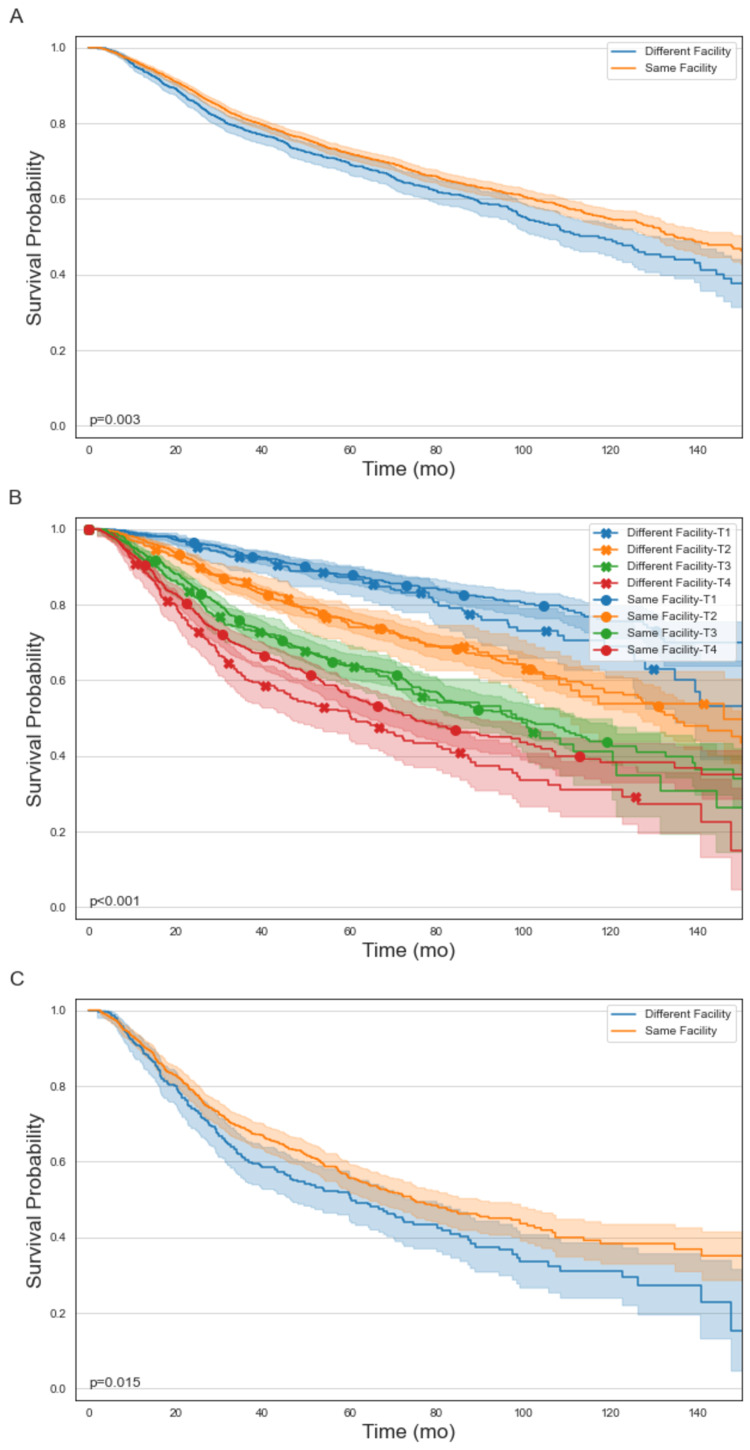
Kaplan-Meier curves of overall survival between patients receiving PORT at the same facility and patients receiving PORT at a different facility as surgery in all patients with salivary malignancies (A), in all patients with salivary malignancies stratified by pathologic T stage (B), and only in patients with pT4 primary salivary malignancies (C)

The median follow-up for all patients was 51 months (range: 2-183 months), and for patients with T4 tumors, the median follow-up was 40 months (range: 2.1-174 months). In the entire cohort, the median OS was 11.2 years in patients who received PORT at the same surgical facility and 9.8 years for patients treated at a different facility (p=0.003) (Figure 2A). However, on multivariable Cox proportional hazards regression, this was not a significant predictor (p=0.055). When stratified by T stage, the survival benefit of receiving PORT at the same surgical facility was only present in T4 tumors (p=0.015) and not in T1 (p=0.071), T2 (p=0.848), or T3 (p=0.451) tumors (Figure 2B, 2C). On subset analysis of patients with T4 tumors, receipt of PORT at the same surgical facility remained associated with an overall survival benefit (HR: 0.78, p=0.011) (Table 3). In this cohort of patients, the median, five-year, and 10-year OS in patients who received PORT at the same versus different facility were 6.2 years, 55.5%, and 38.3% versus 5 years, 50.3%, and 31%, respectively. On multivariable Cox proportional hazards regression, other factors that were associated with improved OS included increasing year of diagnosis (HR: 0.96, p=0.034), age of 65 or younger (HR: 0.61, p<0.001), and treatment at an academic center relative to a Community Cancer Program (HR: 0.56, p=0.002). Histology, margin status, chemotherapy receipt, and time to initiation of radiation were not significant predictors of OS on the initial MVA and therefore were not included in the final MVA. The full MVA is listed in Table 2.

**Table 2:** Multivariate analysis of the predictors of overall survival for patients with primary salivary malignancies HR: hazard ratio; CI: 95% confidence interval; PORT: postoperative radiation therapy; LVSI: lymphovascular space invasion; ECE: extracapsular extension; RT: radiation therapy

	All Patients		T4 Patients	
Characteristic	HR (CI)	p	HR (CI)	p
PORT Location				
Different Facility	Ref	--	Ref	--
Same Facility	0.90 (0.81-1)	0.057	0.78 (0.65-0.95)	0.011
Year of Diagnosis	0.94 (0.92-0.97)	<0.001	0.96 (0.92-1)	0.034
Age (Years)				
≤65	Ref	--	Ref	--
>65	1.91 (1.73-2.11)	<0.001	1.65 (1.38-1.97)	<0.001
Distance to Facility (Miles)				
≤25	Ref	--	Ref	--
>25	1.21 (1.08-1.35)	0.001	1.29 (1.06-1.56)	0.011
Unknown	0.41 (0.31-0.52)	<0.001	0.40 (0.25-0.63)	<0.001
Charlson/Deyo Score				
0	Ref	--	Ref	--
1	1.23 (1.08-1.39)	0.001	1.28 (1.02-1.61)	0.034
2+	1.60 (1.28-1.99)	<0.001	2.10 (1.44-3.06)	<0.001
Hospital Type				
Community Cancer Program	Ref	--	Ref	--
Academic/Research Program	0.80 (0.66-0.97)	0.023	0.56 (0.39-0.81)	0.002
Comprehensive Community Cancer Program	0.86 (0.71-1.05)	0.134	0.65 (0.45-0.94)	0.021
Integrated Network Cancer Program	0.78 (0.62-0.98)	0.035	0.54 (0.35-0.84)	0.006
Unknown	0.38 (0.28-0.53)	<0.001	0.32 (0.18-0.59)	<0.001
ECE				
ECE-	Ref	--	Ref	--
ECE+	1.24 (1.06-1.46)	0.008	1.37 (1.04-1.81)	0.024
Unknown	1.06 (0.91-1.25)	0.449	1.35 (1.02-1.79)	0.036
Pathologic T Stage				
T1	Ref	--	--	--
T2	1.61 (1.37-1.89)	<0.001	--	--
T3	2.04 (1.74-2.39)	<0.001	--	--
T4	2.40 (2.05-2.82)	<0.001	--	--
Pathologic N Stage				
N0	Ref	--	Ref	--
N1	1.68 (1.45-1.94)	<0.001	1.32 (0.99-1.75)	0.060
N2	2.57 (2.27-2.91)	<0.001	1.92 (1.53-2.41)	<0.001
N3	0.87 (0.38-1.96)	0.734	0.77 (0.24-2.45)	0.654
Grade				
Grade 1	Ref	--	Ref	--
Grade 2	1.51 (1.18-1.94)	0.001	1.53 (0.84-2.79)	0.167
Grade 3 or 4	2.86 (2.27-3.59)	<0.001	2.64 (1.50-4.65)	0.001
Unknown	1.92 (1.52-2.43)	<0.001	1.92 (1.08-3.42)	0.026
LVSI				
LVSI-	Ref	--	Ref	--
LVSI+	1.56 (1.35-1.81)	<0.001	1.39 (1.07-1.82)	0.015
Unknown	0.96 (0.80-1.14)	0.617	1 (0.73-1.37)	1.000
Received Chemotherapy				
No	--	--	Ref	--
Unknown	--	--	0.93 (0.53-1.62)	0.796
Yes	--	--	1.21 (1-1.47)	0.050

Among patients with T4 tumors, receipt of PORT at the same facility as surgery was associated with age (<65 versus ≥65: OR: 1.44, p<0.001); being treated at an academic/research center (OR: 1.98, p=0.031), Comprehensive Community Cancer Program (OR: 2.35, p=0.010), or Integrated Network Cancer Program (OR: 2.76, p=0.008) relative to a Community Cancer Program; having either government (OR: 3.97, p=0.012) or private (OR: 3.79, p=0.016) insurance relative to being uninsured; being treated in the Northeast (OR: 1.74, p=0.013) or Midwest (OR: 1.61, p=0.017) relative to the West; and living within 25 miles of the surgical facility (OR: 3.17, p<0.001) (Table 3).

**Table 3:** Logistic regression analysis of the predictors for PORT at the same surgical facility among patients with T4 tumors OR: odds ratio; CI: 95% confidence interval

Characteristic	OR (CI)	p
Age (Years)		
>65	Ref	--
≥65	1.44 (1.44-1.44)	<0.001
Insurance		
Uninsured	Ref	--
Government	3.97 (1.35-11.68)	0.012
Private	3.79 (1.29-11.13)	0.016
Unknown	4.85 (1.01-23.34)	0.049
Hospital Type		
Community Cancer Program	Ref	--
Academic/Research Program	1.98 (1.07-3.67)	0.031
Comprehensive Community Cancer Program	2.35 (1.23-4.50)	0.010
Integrated Network Cancer Program	2.76 (1.30-5.83)	0.008
Region		
West	Ref	--
Midwest	1.61 (1.09-2.37)	0.017
Northeast	1.74 (1.12-2.69)	0.013
South	1.03 (0.61-1.73)	0.916
Distance to Facility (Miles)		
>25	Ref	--
≤25	3.17 (2.34-4.29)	<0.001
Unknown	1.65 (1.04-2.62)	0.035

## Discussion

In this study of patients with primary salivary gland malignancies, we found an association between improved OS and the facility at which the PORT occurred relative to surgery (same versus different), although this appears to primarily be driven by more locally advanced disease, as in patients with T4 primary tumor. On multivariate analysis, when all patients with salivary gland tumors were included, PORT treatment location was not a significant predictor of OS; however, when only T4 patients were included, the location of PORT was significantly associated with OS. This is consistent with more advanced cases requiring more complicated and multidisciplinary care. Margin status was not significantly associated with OS. Of note, over half of the patients did not have PORT initiated within six weeks as recommended by the NCCN, although this was not significantly associated with survival.

The benefit of PORT and surgery being performed at the same facility is not restricted to salivary malignancies. Amini et al. have shown an association between improved survival and congruous PORT/surgical facility in patients with oral cavity squamous cell carcinoma and all head and neck malignancies [8,9]. This has also been shown in studies of patients with head and neck cancers, which have found an association between improved survival and treatment at specialized cancer centers or high-volume centers [3,4,10,11]. This benefit can potentially be attributed to the greater experience and expertise of treating physicians across disciplines and better communication and care coordination within the treatment team. It is worth noting that facility volume was not a significant predictor of survival in the current study. This may be partially explained by the type of facility at which the surgery was performed, where the current study found an association between non-academic cancer centers and decreased survival. This association is potentially related to decreased access to ancillary services at non-academic centers. Indeed, when facility type was included in the multivariate analysis of the location of PORT, it remained significantly associated with OS. Under logistic regression, patients who underwent surgery at community cancer centers were less likely to receive PORT at the same facility compared to patients treated at other types of facilities. Unfortunately, for patients who received PORT at a different facility as their surgery, details on the type of facility where the PORT was received were not available and therefore could not be analyzed. Another interesting contributor to where patients received PORT was insurance type. This supports the conclusion that treatment location is not always patient-driven, which could have ramifications for patient outcomes, particularly in patients with pT4 tumors.

The increase in the overall survival associated with PORT at the same surgery facility is likely due to a number of factors. In our analysis, the advanced pathologic tumor stage appeared to be a driver of the difference in survival. Intuitively, patients with smaller tumors will require less complicated treatment volumes; a well-circumscribed tumor bed measuring ≤2 cm confined to the parotid gland would not be expected to need as much anatomic consideration as a large tumor with perineural invasion and invasion into the adjacent skull. In the latter case, it is essential that the treating radiation oncologist takes into account surgical findings to determine areas most at risk that should be included in treatment volumes. These anatomic considerations are even more pronounced when anatomic distortions are considered. Because of the conformal nature of modern intensity-modulated radiation therapy, missing high-risk areas, such as close or positive margins or perineural invasion requiring nerve tracking, could result in a higher risk of disease recurrence and subsequent disease-related consequences, including death [4]. Although some information required for treatment volume delineation can be obtained from operative notes and pathology reports, contouring for T4 tumors is still very nuanced, requiring direct communication with the surgeon regarding high-risk areas. Such communication is more easily facilitated when both physicians practice within the same facility and, ideally, have more involved input through direct engagement such as within multidisciplinary tumor boards. Although this type of communication cannot be coded in the NCDB, the receipt of surgery and PORT at the same facility offers a plausible surrogate with the aforementioned implications for patient care.

Receipt of surgery and RT at the same facility might also result in more timely administration of adjuvant therapy, with PORT being initiated ≤6 weeks after surgery per NCCN guidelines [1]. The current study did not see an association between surgery/RT facility type and time to adjuvant therapy; however, this association has been noted in prior studies of head and neck cancers [12]. Another relevant factor is total treatment duration. The seminal paper showing treatment duration (time of surgery to end of RT) to be a significant prognostic factor in patients receiving postoperative radiation to the head/neck was limited to mucosal cancers and did not include salivary tumors [13]. A standard course of postoperative radiation should take approximately 6-7 weeks, consistent with the median identified in this study of 46 days. Longer regimens are suggestive of more treatment breaks or logistical issues, which can be related to toxicity, hospitalizations, and transportation, among other factors, and have shown to be associated with decreased overall survival [14]. In this study, a greater proportion of patients who received PORT at their surgical facility had treatment durations of 46 days or shorter. This may be due to improved comprehensive care and supportive services that are more readily available under a single cancer center.

Despite this study benefitting from the large sample size of the NCDB, it also has several associated limitations that are worth noting, namely, the utility of the NCDB is restricted by retrospective analysis as well as variable coding, both in the potential for miscoding and due to limitations in available variables. Therefore, there are potential confounding variables that influence where PORT is received that are not included in the NCDB and therefore cannot be accounted for. The available variables and associated errors with coding also limit more robust characterization of the radiation treatment regimen, most notably with respect to dose, which was not included in the analysis. It is therefore impossible to be confident that all included patients were treated with a standard fractionation schema and whether there were imbalances among patient cohorts. Other missing data include specifics about chemotherapy regimens, pathologic features such as perineural invasion, and more robustly validated comorbidity indices than the number of Charlson comorbidities. Next, facility type in this study is the facility where the surgery was performed; therefore, it is not possible to assess the type of facility where patients received PORT if they were not treated at the same facility, and the influence of that facility type on survival cannot be assessed. Lastly, the NCDB only provides data on OS and does not permit analysis of other pertinent oncologic outcomes such as progression-free survival, local control, and disease-specific survival.

## Conclusions

To our knowledge, this is the first study to identify an association between improved survival and receipt of PORT at the same surgical facility in patients with locally advanced salivary gland cancers. The impact is initially small and increased with time, with improvements in the five-year OS of about 4% and 10-year OS of 7%. These data suggest that recommending patients with more advanced diseases to receive adjuvant treatment at the same surgical facility is reasonable. Future research is warranted to better characterize the causes and magnitude of these differences to better counsel patients on optimal treatment management.
